# NDS27 combines the effect of curcumin lysinate and hydroxypropyl-β-cyclodextrin to inhibit equine PKCδ and NADPH oxidase involved in the oxidative burst of neutrophils

**DOI:** 10.1016/j.fob.2014.11.004

**Published:** 2014-11-18

**Authors:** Sandrine Derochette, Ange Mouithys-Mickalad, Thierry Franck, Simon Collienne, Justine Ceusters, Ginette Deby-Dupont, Philippe Neven, Didier Serteyn

**Affiliations:** a Center for Oxygen, R&D (CORD), Institute of Chemistry, B6a, University of Liège, Allée du 6 Août 13, B-4000 Liège, Belgium; b Faculty of Veterinary Medicine, Equine Clinic, B41, University of Liège, Boulevard de Colonster 20, B-4000 Liège, Belgium; c Department of Physics, Biomedical Spectroscopy, B5a, University of Liège, Allée du 6 Août 17, B-4000 Liège, Belgium; d Faculty of Pharmacy, Laboratory of Medicinal Chemistry, B36, University of Liège, Avenue de l’Hôpital, 1, B-4000 Liège, Belgium

**Keywords:** γ-CD, γ-cyclodextrin, fMLP, formyl-methionyl-leucyl-phenylalanine, HPβCD, hydroxypropyl-β-cyclodextrin, MPO, myeloperoxidase, PMNs, polymorphonuclear neutrophils, ROS, reactive oxygen species, Curcumin lysinate, Cyclodextrin, Inflammation, NADPH oxidase, PKC

## Abstract

Polymorphonuclear neutrophils (PMNs) are involved in host defence against infections by the production of reactive oxygen species (ROS), but excessive PMN stimulation is associated with the development of inflammatory diseases. After appropriate stimuli, protein kinase C (PKC) triggers the assembly of NADPH oxidase (Nox2) which produces superoxide anion (O_2_^•^^−^), from which ROS derive. The therapeutic use of polyphenols is proposed to lower ROS production by limiting Nox2 and PKC activities. The purpose of this study was to compare the antioxidant effect of NDS27 and NDS28, two water-soluble forms of curcumin lysinate respectively complexed with hydroxypropyl-β-cyclodextrin (HPβCD) and γ-cyclodextrin (γ-CD), on the activity of Nox2 and PKCδ, involved in the Nox2 activation pathway. Our results, showed that NDS27 is the best inhibitor for Nox2 and PKCδ. This was illustrated by the combined effect of HPβCD and curcumin lysinate: HPβCD, but not γ-CD, improved the release of curcumin lysinate and its exchange against lipid or cholesterol as demonstrated by the lipid colouration with Oil Red O, the extraction of radical lipophilic probes recorded by ESR and the HPLC measurements of curcumin. HPβCD not only solubilised and transported curcumin, but also indirectly enhanced its action on both PKC and Nox2 activities. The modulatory effect of NDS27 on the Nox2 activation pathway of neutrophils may open therapeutic perspectives for the control of pathologies with excessive inflammatory reactions.

## Highlights

• The antioxidant effects of curcumin lysinate complexed with two cyclodextrins were compared.
• NDS27 is complexed with hydroxypropyl-β- and NDS28 with γ-cyclodextrin.
• NDS27 but not NDS28 inhibits translocation and activity of PKCδ and NADPH oxidase.
• NDS27 but not NDS28 improved the release of curcumin lysinate and its exchange with membrane lipids.
• NDS27 is a good candidate molecule to inhibit ROS production by neutrophils.

## 1 Introduction

For the destruction of pathogenic agents during phagocytosis, polymorphonuclear neutrophils (PMNs) increase their molecular oxygen consumption, a process known as the “oxidative burst”, to produce reactive oxygen species (ROS) [1]. During this oxidative burst, NADPH oxidase, or Nox2, a multi-component key enzyme, produces superoxide anion O_2_^•^^−^, the first specie from which most of the other ROS derive. In resting cells, subunits of NADPH oxidase are spread between membrane and cytosol. Upon stimulation of appropriate receptor, such as C5a or formyl-methionyl-leucyl-phenylalanine (fMLP) receptor, translocation of the cytosolic Nox2 components p40phox, p47phox and p67phox to the membrane-bound flavocytochrome b_558_ (comprising gp91phox and p22phox) is induced [2]. This translocation requires, not only a conformational modification and phosphorylation of the Nox2 components, but also a complex set of protein/protein interactions [3]. The protein kinase C (PKC) family appears to play a major role in this process especially upon stimulation of PMNs by phorbol 12-myristate 13-acetate (PMA) or fMLP [2].

Neutrophils express PKCα, β and δ that can all initiate the superoxide generation in cell-free systems. PKCδ is required after fMLP stimulation and needs phosphatidylserine and diacylglycerol but is Ca^2+^ independent unlike other PKC family members [4]. PKCs δ are primarily localised in the cytosol of unstimulated neutrophils. In response to agonist stimulation, a fraction of PKCs δ rapidly becomes membrane-associated and this change of localisation is required to activate NADPH oxidase by phosphorylation of its cytosolic and membrane subunits, especially p47phox [5,6].

The oxidant activity of neutrophils is required for their normal microbicidal function but their excessive stimulation, as found in inflammation situations, leads to the extracellular release of ROS and oxidant enzymes such as myeloperoxidase (MPO), causing deleterious effects on neighbouring cells and tissues [7]. As in humans, uncontrolled activation of PMNs in the horse can be related to irreversible endotoxic shock [8–10]. A therapeutic goal could be to lower the oxidant activity of excessively stimulated neutrophils by modulating the activity of MPO [11] or NADPH oxidase, both responsible for ROS production. To this end, NADPH oxidase appears as the prime target since it produces the first ROS from which the others are derived [12].

With this objective in mind, we were interested in the study of curcumin, a natural phenolic compound of turmeric (*Curcuma longa*), widely used as a food dye and flavouring [13]. Curcumin was reported to inhibit the activation of leukocytes, including PMNs [11,14] and monocytes [15], and to have numerous other properties [13,14,16–18]. This wide range of biological activities, associated with a low toxicity justifies the therapeutic interest in using curcumin [14,19]. The action of curcumin involves interactions with transcription factors, cytokines and enzymes [16,20] and inhibition of the oxidative response by activated PMNs through the inhibition of ROS generation and scavenging [14,21]. Curcumin can also interfere with the MPO activity [11], a major contributor to ROS production in neutrophils. However, as the insolubility of curcumin in aqueous milieu makes its use very difficult in biochemical assays, we developed two highly water-soluble salts of curcuminoid derivative, NDS27 and NDS28, complexed with hydroxypropyl*-*β*-*cyclodextrin (HPβCD) and γ-cyclodextrin (γ-CD) respectively [22]. We previously demonstrated the capacity of curcumin to inhibit the oxidative burst of neutrophil through the inhibition of MPO [11] and NADPH oxidase [23] activities. Furthermore, we have also shown that, this capacity appeared to be improved in the soluble form of curcumin lysinate via its ability to interact with membranes and to enter the cells [24]. We thus hypothesised that the cyclodextrin from NDS27, HPβCD, enhances the action of curcumin against the oxidative burst. The purpose of the present work was to determine and compare the effect of the complexes of curcumin lysinate with two different types of cyclodextrins (HPβCD and γ-CD) on the activity of NADPH oxidase and PKCδ situated just upstream in the PMN activation pathway.

## 2 Materials and methods

### 2.1 Chemicals and reagents

Analytical grade Na, K, Ca, Mg salts, polyvinylidene difluoride (PVDF) membrane and Tween 20 were from Merck. Phenylmethylsulfonyl fluoride (PMSF), leupeptin, pepstatin, guanosine 5′-[γ-thio] triphosphate tetralithium salt (GTP-γ-S), sodium arachidonate, cytochrome C from horse heart, nicotinamide adenine dinucleotide phosphate reduced (NADPH), superoxide dismutase from bovine erythrocyte (SOD), cytochalasin B, N-formyl-methionyl-leucyl-alanine (fMLP), Oil Red O, ethylene glycol-bis(2-aminoethylether)-N,N,N′,N′-tetraacetic acid (EGTA), *N*_α_-tosyl-l-lysine chloromethyl ketone hydrochloride, HEPES, 5-doxyl stearic acid (2-(3-carboxypropyl)-4,4-dimethyl-2-tridecyl-3-oxazolidinyloxy, free radical) and 16-doxyl stearic acid (2-(14-carboxytetradecyl)-2-ethyl-4,4-dimethyl-3-oxazolidinyloxy, free radical) were purchased from Sigma–Aldrich (Bornem, Belgium). Nitrotetrazolium blue chloride (NBT) and 5-bromo-4-chloro-3-indolyl phosphate disodium salt (BCIP) were both from Fermentas (Sankt Leon-Rot, Germany) and IMDM medium, penicillin, streptomycin, amphotericin B, fetal bovine serum and Hanks’ balanced salt solution (HBSS) were from Gibco-Invitrogen (Fischer Scientific, Erembodegem, Belgium). Isopropanol was purchased from VWR (Leuven, Belgium). Bovine serum albumin (BSA) was from Roche (Mannheim, Germany). NDS27, NDS28, HPβCD and γ-CD were gifts from Philippe Neven (Faculty of Pharmacy, University of Liège, Belgium). All concentrations of NDS27 and NDS28 were expressed in term of molar concentration of pure curcumin and contain a 5-fold increased concentration in HPβCD and γ-CD respectively.

### 2.2 Methods

#### 2.2.1 Animals experiments

All the experiments were realised in accordance with the ethic committee of the Faculty of Veterinary Medicine of University of Liege (agreement number 1474). Equine neutrophils were isolated from whole blood drawn on citrate–phosphate–dextrose (PL146, Baxter Healthcare) or EDTA (Vacuette, Greiner Bio-one) from the jugular vein of healthy horses, fed and bred in identical conditions and not under medical treatment (Faculty of Veterinary Medicine, University of Liège, Belgium). Horses were considered as healthy if no clinical signs of acute disease were observed at the time of sampling. Each batch of neutrophils was obtained from blood drawn from one horse. Samples were collected by a qualified veterinarian from the equine clinic of University of Liège in aseptic conditions without any surgery, anaesthetic procedure or risk for the animal.

#### 2.2.2 Cell culture

Human promyelocytic leukemia cells (HL-60) were obtained from the American Type Culture Collection (ACCT, USA) and cultured in IMDM medium supplemented with 20% (v/v) fetal bovine serum, 100 units/ml penicillin/streptomycin, 1.25 mg/ml amphotericin B, and 2 g/l NaHCO_3_ in 50 ml flasks at 37 °C in a 5% CO_2_ humidified atmosphere. Cells were supplied with fresh medium two to three times per week to maintain log phase growth. Once a week, the contents of culture flasks were centrifuged and the cell pellets resuspended in fresh IMDM and divided into new culture flasks. Before each experiment, cells were counted with Burker’s cell (Briare, France) to reach the cell number required for the experiments.

#### 2.2.3 Detection of phosphorylated PKC by Western blot

Equine neutrophils (20 × 10^6^ cells) isolated as previously described [24], were resuspended in 1 ml HBSS with 10^−4^ M NDS27 or NDS28 or 5 × 10^−4^ M HPβCD or γ-CD. A control with HBSS alone was realised. After 30 min incubation with the appropriate treatment at 37 °C, cells were activated with cytochalasin B (CB, 5 μM) for 15 min followed by another 15 min incubation with 10^−8^ M fMLP. The suspensions were centrifuged (10 min, 450×*g*) to remove NDS27, NDS28, HPβCD or γ-CD and cells were resuspended in 3 ml of Buffer A (HEPES 10 mM, KCl 100 mM, NaCl 10 mM, MgCl_2_ 3.5 mM, EGTA 12 mM, pH 7.3) [25] supplemented with a protease inhibitor cocktail (N-α-p-tosyl-l-lysine chloromethyl ketone 10 mM, PMSF 1 mM, leupeptin 1.8 μM, pepstatin 1.5 μM). Thereafter cells were sonicated (6 × 5 s, 30% amplitude, Labsonic P, Sartorius) and ultracentrifuged (1 h, 165,000×*g*, 4 °C). The supernatant, corresponding to cytosolic proteins, and the pellet containing membrane proteins resuspended in 500 μl of Buffer A supplemented with anti-proteases cocktail, were kept for further western blotting analysis.

Protein samples (5–10 μg in a total volume of 10 μl Buffer A) were added with 4 μl of loading buffer (Nu PAGE LDS sample buffer, Invitrogen) and 1.6 μl of reducing agent (Nu PAGE sample reducing agent, Invitrogen). Samples were denatured 10 min at 70 °C and 15 μl were loaded on a 4–12% Bis–Tris gel (Nu PAGE, Invitrogen). After electrophoresis at 200 V for 1 h with MOPS SDS Running Buffer (Invitrogen), the proteins were transferred to a PVDF membrane using a semi-dry electroblotter (55 min, 15 V) and then blocked 30 min in Tris-buffered saline (TBS) containing Tween 20 (0.1%) and BSA (1%). After washing with TBST (TBS + 0.1% Tween 20), the membranes were incubated for 1 h with p-PKC δ rabbit polyclonal IgG (Santa Cruz Biotechnology, pPKCδ ser 645-R, sc18369-R, dilution: 200×), washed once with TBST, then incubated for 30 min with anti-rabbit IgG antibodies from goat conjugated to alkaline phosphatase (Abcam, ab97048, dilution: 3000×). After three washing steps of 5 min each with TBST and another one with TBS, the staining was achieved by using Nitro blue tetrazolium (NBT) and 5-Bromo-4-chloro-3-indolyl phosphate (BCIP) as substrate for alkaline phosphatase. To quantify the blot, imageJ software [26] was used. The relative values were reported by mg of proteins and the ratio between p-PKCδ found in membrane and in the cytosol was calculated to evaluate the part of PKCδ that migrated to the membrane.

#### 2.2.4 IC_50_ determination of NDS27 and NDS28 on PKCδ activity

A radiometric protein kinase assay (^33^PanQuinase Activity assay) made by ProQinase (Freiburg, Germany) was used for measuring the kinase activity of the PKCδ protein kinase. The kinase assay was performed in 96-well FlashPlates from PerkinElmer (Boston, MA, USA) in a 50 μl reaction volume. The reaction cocktail was prepared in the following order: 20 μl of assay buffer (or standard buffer), 5 μl of ATP solution (in H_2_O), 5 μl of tested compound (NDS27 and NDS28 from 10^−4^ M to 3 × 10^−9^ M; HPβCD and γ-DC from 5 × 10^−4^ to 1.5 × 10^−8^ M in 10% DMSO) and 20 μl enzyme/substrate mix. The assay mixture contained 70 mM HEPES–NaOH, pH 7.5, 3 mM MgCl_2_, 3 mM MnCl_2_, 3 μM Na-orthovanadate, 1.2 mM DTT, 50 μg/ml PEG_20000_, 10 μM ATP, [γ-^33^P]-ATP (approximatively 6 × 10^5^ cpm per well), 4.5 nM recombinant PKCδ (ProQinase), 0.125 μg/50 μl substrate (PKC 19–31).

The reaction cocktails were incubated at 30 °C for 60 min. The reaction was stopped with 50 μl of 2% (v/v) H_3_PO_4_ and the plates were washed two times with 200 μl of 0.9% (w/v) NaCl. Incorporation of ^33^Pi was determined with a microplate scintillation counter (Microbeta, Wallac). All assays were performed with a Beckman Coulter/SAGIAN core system.

The median value of the counts obtained in absence of a protein kinase but in presence of the substrate was defined as “low control” and represented the unspecific binding of radioactivity to the plate. The median value of the counts for the protein kinase fully active without inhibitor was taken as the “high control”. The difference between high and low control was taken as 100% activity. The residual activity for each tested compound was calculated with the following formula:

Residual Activity(%)=100×[(cpm of compound-low control)/(high control-low control)]

The residual activities for each concentration and the compound IC_50_ values were calculated using Quattro Workflow V3.1.0 software (Quattro Research GmbH, Munich, Germany, www.quattro-research.com). The fitting model for IC_50_ determinations was “Sigmoidal response (variable slope)” with parameters “top” fixed at 100% and “bottom” at 0%. The fitting method used was a least-square fit.

#### 2.2.5 Cell-free system of oxidase activation

As previously described [23], 2 μg of crude membranes and 50 μg of cytosolic proteins were stimulated by addition of 10 μl sodium arachidonate and 2 μl of guanosine 5′-[γ-thio] triphosphate tetralithium salt (GTP-γ-S) (2 μM). A preliminary assay was performed with each batch to determine the optimal amount of sodium arachidonate to be added to provide maximal oxidase activation. The cell-free assay activation was performed in 96-well plates (microtiter assembly breakable strip, Thermo Fisher Scientific) in a total volume of 200 μl phosphate buffered saline (PBS) plus 10 mM MgSO_4_. After 5 min incubation under stirring (RT, 600 rpm), 30 μl cytochrome C (100 μM) were added. Reduction of cytochrome C was initiated by the addition of 10 μl NADPH (250 μM) and the increase of absorbance at 550 nm was followed with a multi-plate reader (Multiskan Ascent FL, Thermo Fisher Scientific) during 5 min. For each experiment, a control supplemented with 200 U/ml superoxide dismutase (SOD), was performed in parallel to the same test without SOD (replaced by an equivalent volume of PBS + MgSO_4_). The difference of absorbance between the reduction of cytochrome C in the presence and absence of SOD was used to evaluate the specific reduction of cytochrome C by O_2_^•^^−^ generated by the oxidase activity. The O_2_^•^^−^ concentration was calculated using Beer–Lambert law with an absorption coefficient of cytochrome C equal to 21.1 mM^−1^ cm^−1^ at 550 nm.

In a first experimental model, the tested molecules (10^−6^–10^−4^ M NDS27 or NDS28 and 5 × 10^−6^ to 5 × 10^−4^ M HPβCD or γ-CD) were pre-incubated for 5 min at room temperature under stirring before the assembly of NADPH oxidase by sodium arachidonate and GTP-γ-S. Then, cytochrome C, SOD (for controls) or PBS + MgSO_4_ and NADPH were added to measure the oxidase activity. In a second model, the tested molecules were added under stirring (600 rpm) after the assembly with sodium arachidonate and GTP-γ-S and, after 5 min incubation, the NADPH oxidase activity was measured as for the first model.

#### 2.2.6 Measurement of lipid content by Oil Red O

Five million PMNs/milliliter in HBSS were incubated with 10^−3^, 2 × 10^−3^ or 3 × 10^−3^ M NDS27 or NDS28 or with 5 × 10^−3^, 10 × 10^−3^ or 15 × 10^−3^ M HPβCD or γ-CD for 2 h at 37 °C. Cells were then centrifuged (5 min, RT, 524×*g*) and the supernatant containing the excess of tested molecules, was discarded. The pellet was washed with 1 ml of HBSS. After the washing step, the cell pellets were separated in two parts: in the first one, 500 μl of Oil Red O (1% w/v diluted in a mixture of isopropanol and water 3:2) were added and in the second part, 500 μl of isopropanol/water mixture (3:2) were added as control. After a 30-min period of incubation at 37 °C, cells were washed three times with 1 ml of HBSS and resuspended in 500 μl of HBSS. 200 μl of each sample were placed in the cavities of a microplate and the absorbance was measured at 540 nm with a microplate reader (Multiskan Ascent FL, Thermo Fisher Scientific). Absorbance values of the control tests with isopropanol/water mixture were subtracted from absorbance values of cells coloured by Oil Red O to avoid interferences due to the natural colour of curcumin.

#### 2.2.7 Electron spin resonance (ESR) experiments with 5- and 16-DSA

HL-60 cells (5 × 10^6^/500 μl of HBSS) were incubated for 20 min with 5 μl of either 5- or 16 DSA (0.01 M). The cells were then centrifuged and the pellets were resuspended in 500 μl HBSS supplemented with either 10^−4^ M NDS27, NDS28, 5 × 10^−4^ M HPβCD or γ-CD and incubated for 10 min at RT. Subsequently, cells were centrifuged and the pellet (resuspended in 500 μl of HBSS) and supernatant were transferred into separate micro-caps tubes (sealed with rubber and transferred into ESR tube), placed into the cavity of the ESR spectrometer. The ESR characteristic spectra of the 5-DSA and 16-DSA signals were recorded. The measurements were implemented at 300 K with a continuous wave spectrum EMX-micro of Bruker (Brüker, Rheinstetten, Germany), operating at fixed X-band frequency of 9.5 GHz and at a microwave power of 10.88 mW. The instrumental settings were as follows: 100 kHz modulation frequency, 2 G modulation amplitude, 3389.698 G magnetic field center, and 3.56 × 10^4^ receiver gain. The sweep width was 89.652 G and the total number of scans was five.

#### 2.2.8 HPLC quantification of curcumin from NDS27 and NDS28 incorporated into the cells

HPLC quantification of curcumin was performed as previously described [24]. Briefly, 100 × 10^6^ isolated PMNs were resuspended in 2 ml of PBS supplemented with NDS27 or NDS28 (10^−4^ M) and incubated 30 min at 37 °C. The cells were then washed and separated into different fractions [24]. The assay of curcumin was performed by a HPLC system including a Merck Hitachi L-7100 solvent delivery system (pump) working at a flow rate of 0.7 ml/min, a Merck Hitachi L-7400 UV detector and a 7725i rheodyne injector with a loop of 20 μl. The EZ Chrom ELITE software was used for integration. Chromatography was conducted using a Merck LichroCart 125-4 LiChrospher 100 RP-18 (5 μm) column with an isocratic elution with tetrahydrofuran (THF)/50 mM citrate buffer (pH = 6) (66:34) and detection at 419 nm using a UV detector.

### 2.3 Statistical analysis

Data are given in relative values (%) in reference to control groups defined as 100%. All data are expressed as mean ± standard error of the mean (S.E.M.) of at least two independent experiments made with different cell batches. Statistical analysis was realised with corresponding solvent vehicle control group as reference. An independent *t*-test was performed with SOFA statistics 1.3.2 (Released under open source AGPL3 licence© 2009–13 Paton-Simpson & Associates Ltd.). A Mann–Whitney test was realised for data that did not respect a Gaussian distribution. A *p*-value <0.05 was considered as significant.

## 3 Results

### 3.1 Effect of NDS27 and NDS28 on the phosphorylation and translocation of PKCδ

**Fig. 1:**
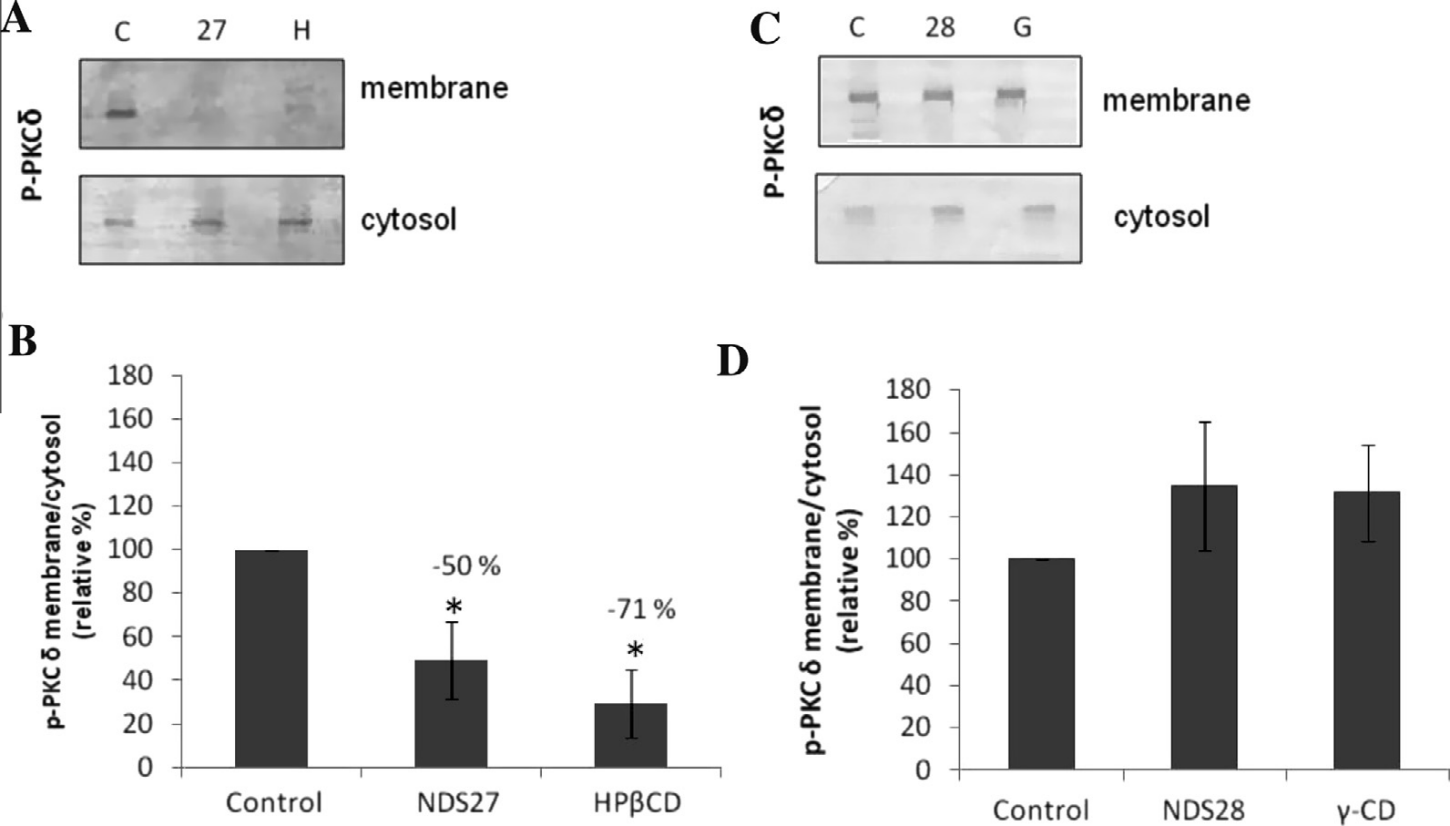
Detection of phosphorylated PKCδ (p-PKCδ) in cytosolic and membrane extracts from activated PMNs treated or not with NSD27, NSD28, HPβCD or γ-CD. (A and C) Example of western blotting. C: control cells; 27: cells treated with 10^−4^ M NDS27; H: cells treated with 5 × 10^−4^ M HPβCD; 28: cells treated with 10^−4^ M NDS28; G: cells treated with 5 × 10^−4^ M γ-CD. (B and D) Quantification of pPKC detected in the cellular extracts (ImageJ software). For each condition, results are expressed as the ratio of p-PKCδ detected in membrane vs. cytosolic extracts. The percentage of inhibition indicated on the top of each column is calculated vs. the ratio obtained for control cells taken as 100%. Control: control cells; NDS27: cells treated with 10^−4^ M NDS27; HPβCD: cells treated with 5 × 10^−4^ M HPβCD; NDS28 cells treated with 10^−4^ M NDS28; γ-CD: cells treated with 5 × 10^−4^ M γ-CD. ^∗^*p* < 0.05 vs. control. Data are given as means ± S.E.M. (*n* ⩾ 4).

The Western blot analysis showed that, in activated PMNs (C), the signal corresponding to p-PKCδ was stronger in the fraction containing the membranes compared to cytosolic fractions (Fig. 1A). Upon pre-incubation of the cells with 10^−4^ M NDS27 or with 5 × 10^−4^ M HPβCD, we observed a reduction of the p-PKCδ content in membranes but an increase in the cytosol suggesting that NDS27 and HPβCD decreased the migration of phosphorylated PKC to the membrane and thus the enzyme activation (Fig. 1A). The quantification and calculation of p-PKCδ ratio between membrane and cytosol extracts showed that NDS27 and HPβCD significantly reduced the PKCδ activation (respectively −50% and −71%, Fig. 1B). Incubation of PMNs with 10^−4^ M NDS28 or 5 × 10^−4^ M γ-CD slightly increased the p-PKCδ content in membrane fractions and reduced it in the cytosol (Fig. 1C), resulting in a non-significant increase of the membrane/cytosol ratio (Fig. 1D).

### 3.2 Effect of NDS27 and NDS28 on PKCδ activity

**Fig. 2:**
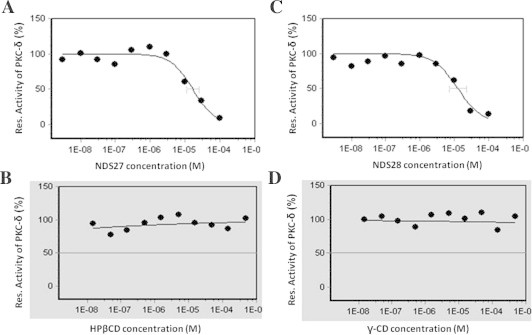
Effect of NDS27 (A), HPβCD (B), NDS28 (C) or γ-CD (D) concentration (black lines) on the residual activities of PKCδ (%). A horizontal light gray line shows the inflection points of the curves corresponding to the IC_50_.

The hill slopes characteristic of the curve shapes were calculated for each condition and a hill slope higher than −0.4 would mean that the curve was not sigmoidal, very flat or not descending. As observed in Fig. 2A and C, the shape of the dose–response curves with NDS27 and NDS28 were sigmoidal as confirmed by the hill slopes lower than −0.4 (−1.37 and −1.23 respectively). The IC_50_ determined for the two molecules were quite similar: 1.67 × 10^−5^ M for NDS27 and 1.26 × 10^−5^ M for NDS28. For HPβCD and γ-CD the hill slopes were higher than −0.4 showing no inhibition (Fig. 2B and D).

### 3.3 Effect of NDS27 and NDS28 on the activity of NADPH oxidase

**Fig. 3:**
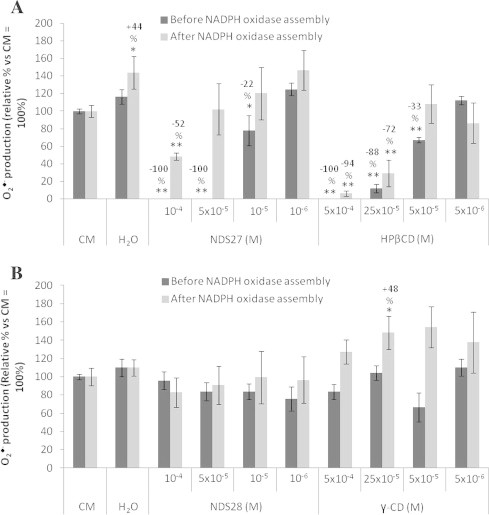
Effect of NDS27 and HPβCD (A) or NDS28 and γ-CD (B) on NADPH oxidase assembly and activity as determined by O_2_•^−^ production in cell-free assay. The tested molecules were added before (dark gray) or after (light gray) NADPH oxidase assembly. The percentages of inhibition indicated on the top of each column were calculated vs. the respective control group (CM), ^∗∗^*p* < 0.001, ^∗^*p* < 0.05. CM: reconstituted complex without the tested molecule. Data are given as means ± S.E.M (*n* ⩾ 6).

NDS27, NDS28, HPβCD or γ-CD were added before or after NADPH oxidase activation with GTP-γ-S and sodium arachidonate. NDS27 exhibited a dose-dependent inhibitory effect on the O_2_^•^^−^ production and this effect was more pronounced when the molecule was added before the enzyme assembly (Fig. 3A). The inert vehicle substance, or excipient of NDS27, HPβCD, has a stronger inhibitory effect when added alone than in combination with curcumin salt (NDS27). Again, the effect of HPβCD was more important when it was added before the NADPH oxidase activation. NDS28 and γ-CD added before the enzyme assembly had no effect on the NADPH oxidase activity (Fig. 3B). When γ-CD was added after assembly, it showed a pro-oxidant activity by increasing O_2_^•^^−^ production, however a significant effect was only observed at 25 × 10^−5^ M.

### 3.4 Influence of NDS27 and NDS28 on the lipid content of cell membranes

**Fig. 4:**
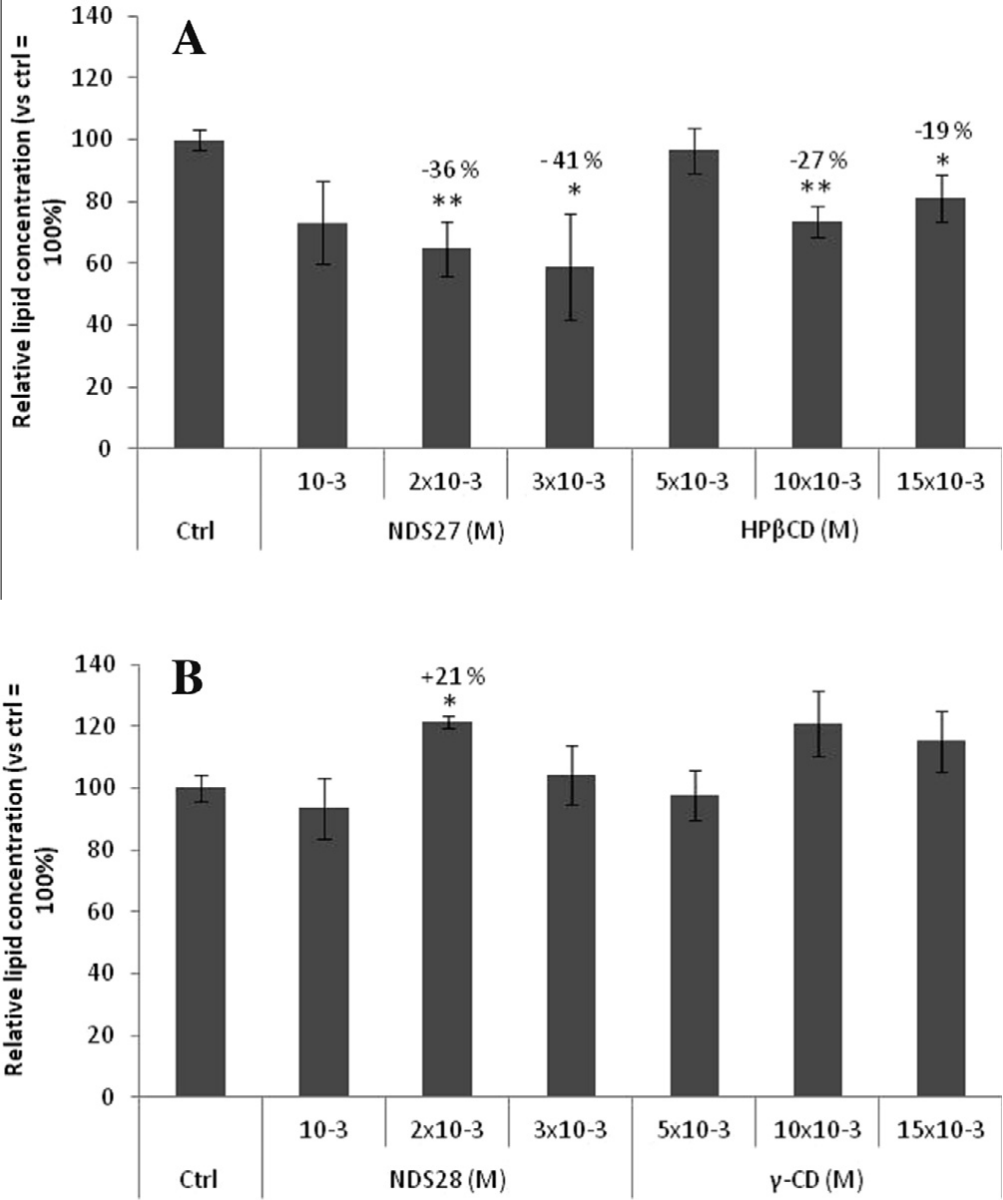
Effect of NDS27 and HPβCD (A) or NDS28 and γ-CD (B) on the lipid content of PMNs measured by Oil Red O colouration and compared to control cells. The percentages of inhibition indicated on the top of each column were calculated vs. cells control group (Ctrl), ^∗∗^*p* < 0.001, ^∗^*p* < 0.05. Data are given as means ± S.E.M (*n* ⩾ 6).

Pre-incubation with NDS27 (2 and 3 × 10^−3^ M) resulted in a significant dose-dependent reduction in the lipid content of PMNs (Fig. 4A). HPβCD alone showed the same significant effect at the final corresponding concentration of 10 × 10^−3^ and 15 × 10^−3^ M but the reduction of lipid content was less important than observed with NDS27. In contrast, pre-incubation with both NDS28 and γ-CD did not affect the lipid content of the cells, except at 2 × 10^−3^ M, where we observed an increase of Oil Red O colouration (Fig. 4B).

### 3.5 Effect of NDS27 and NDS28 on 5-DSA and 16-DSA probe extraction from cell membranes

**Fig. 5:**
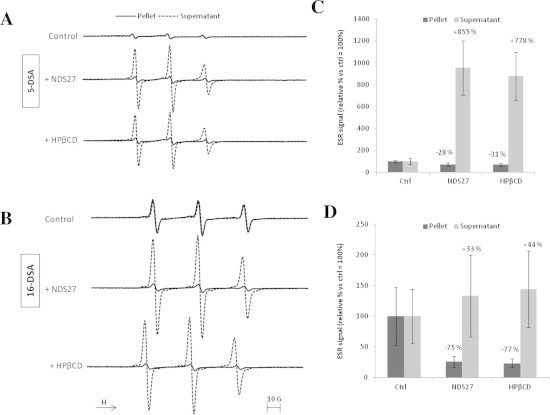
Example of ESR spectra obtained with 5 × 10^6^ HL-60 cells incubated with 5-DSA (A) or 16-DSA (B) and treated with 10^−4^ M NDS27 or 5 × 10^−4^ M HPβCD. After cells centrifugation, the ESR spectra were recorded on the supernatant (dotted lines) and on the pellets (plain lines) reconstituted in HBSS. Reproducible results were obtained in at least three replicate experiments. The signal intensities of 3-lines ESR spectra, corresponding to 5-DSA spectra (C) or 16-DSA spectra (D) respectively, were calculated and expressed in relative percentage values vs. control ones taken as 100%. Data are given as means ± S.E.M (*n* ⩾ 3).

**Fig. 6:**
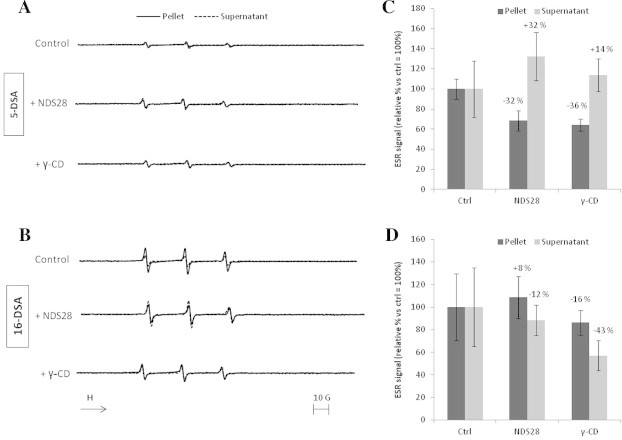
Example of ESR spectra obtained with 5 × 10^6^ HL-60 cells incubated with 5-DSA (A) or 16-DSA (B) and treated with 10^−4^ M NDS28 or 5 × 10^−4^ M γ-CD. After cells centrifugation, the ESR spectra were recorded on supernatant (dotted lines) and on pellets (plain lines) reconstituted in HBSS. Reproducible results were obtained in at least three replicate experiments. The signal intensities of 3-lines ESR spectra, corresponding to 5-DSA spectra (C) or 16-DSA spectra (D) respectively, were calculated and expressed in relative percentage values vs. control ones taken as 100%. Data are given as means ± S.E.M (*n* ⩾ 3).

The 5-DSA and 16-DSA probes are detected by ESR spectroscopy based on their nitroxide radical located on 5th and 16th carbon, respectively. They are characterised by different lengths of the hydrophobic chain near the nitroxide radical allowing them to anchor more (16-DSA) or less (5-DSA) deeply in the membrane. The ESR spectra of both 5-DSA and 16-DSA probes, incubated with HL-60 cells (control), were quite similar for the cellular pellet and the supernatant, meaning that only a part of the probe is fixed on the cells after incubation (Figs. 5A and B and 6A and B). When the cells pre-incubated with 10^−4^ M NDS27 or 5 × 10^−4^ M HPβCD, increased ESR signal was observed for the supernatant (Fig. 5A, dotted lines), which is characteristic of the ESR spectrum shape of 5-DSA free in a buffer medium. The spectrum recorded on the pellets showed weak signals compared to control pellets (Fig. 5A, plain lines) suggesting that only a small part of the probe remained in the cell membrane after NDS27 or HPβCD treatment. The quantification of the ESR signal intensity allowed us to establish the tight relationship between the presence of the drug (e.g. NDS27) within the cell membrane and the uptake of the probe (5-DSA) outside, thus increasing the signal intensity of free 5-DSA from 895% for NDS27 vs. 778% in the presence of HPβCD (Fig. 5C). We also obtained a reduction of the signal intensity for the cellular pellets: −28% for NDS27 and −30% for HPβCD (Fig. 5C). A similar effect was observed when using the 16-DSA probe instead of the 5-DSA one. Intense signals, characteristic of the free probe, observed in the supernatant when cells were pre-incubated with NDS27 or HPβCD (Fig. 5B) but the difference for both signals compared to the control was less marked than with 5-DSA. The quantification of signal intensities showed an increase of 58% and 44% respectively in the supernatant for NDS27 and HPβCD and a reduction of 74% (NDS27) and 77% (HPβCD) in cellular pellets compared to control (Fig. 5D). On the contrary, NDS28 and γ-CD, exhibited little influence on the uptake of 5-DSA and 16-DSA outside the cell membrane compared to control (Fig. 6).

### 3.6 Capacity of curcumin from NDS27 and NDS28 to enter the cells

**Fig. 7:**
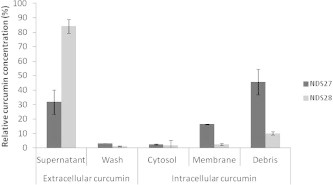
Quantification of curcumin from NDS27 or NDS28 (10^−4^ M) that entered or not into PMNs after incubation. Supernatant: curcumin found in the supernatant after 30 min incubation with cells. Wash: curcumin recovered after two washings of the cells. Cytosol and membrane: curcumin found in cytosolic and membrane fractions respectively after ultracentrifugation of sonicated cells. Debris: curcumin found in granules, unbroken cells and nuclei. Data are expressed in relative percentages vs. the sum of the amounts of curcumin found in the different fractions taken as 100% and are given as the mean ± S.E.M of two (for NDS27) or six (for NDS28) separate experiments (*n* ⩾ 2).

We previously published that the supernatant and the wash fractions of PMNs incubated with NDS27 contained 31.80% and 3.19% of total curcumin respectively. After sonication and two centrifugation steps, 2.48% of curcumin was found in the cytosol, 16.49% in the membrane and 45.65% in the cellular debris, consisting of granules, nuclei and unbroken cells. The total relative amount of curcumin entering the PMNs was estimated to be 64.62% [24]. Our HPLC measurement also demonstrated that most of the curcumin from NDS28 did not enter PMNs incubated with this molecule as 84.22% of curcumin remained in the supernatant and 1.17% was recovered after the two washing steps. After sonication and two centrifugation steps, 1.78% of curcumin was found in the cytosol fraction, 2.63% in the membranes and 10.07% in the cellular debris (Fig. 7).

## 4 Discussion

Our present findings [11] and those previously described by other groups including Nakamura et al. [27] have successfully shown the ability of curcumin to inhibit the ROS production mediated by stimulated neutrophils. More recently, our group brought further evidence that curcumin acts not only by a ROS-scavenging effect, but also by inhibiting the NADPH oxidase activity in cell-free assay [23]. However, the lack of curcumin solubility in aqueous medium combined with a poor intestinal absorption [28] may reduce its potential therapeutic effects and complicate its use for biochemical assays. For these reasons, our team has synthesised NDS27 and NDS28, two water-soluble complexes of curcumin lysinate prepared with two different cyclodextrins: 2-hydroxypropyl-beta-cyclodextrin (HPβCD) and γ-cyclodextrin (γ-CD), respectively. As for curcumin, both NDS27 [24] and NDS28 (unpublished results) presented great efficiency in inhibiting ROS production from activated PMNs and also led to a reduction of the activity of MPO, a strong oxidant enzyme of PMNs. However, their effects on NADPH oxidase activity, more and more considered as a target to modulate inflammatory events [12], had not yet been investigated.

In this study, we demonstrate that NDS27 behaves as a potent inhibitor of NADPH oxidase in cell-free system. The inhibitory effect was more efficient when NDS27 was added before (significant inhibition from 10^−5^ M) than after the enzyme assembly (significant inhibition at 10^−4^ M), suggesting that NDS27 acts by inhibiting the enzyme assembly rather than by acting on its activity. These findings are in accordance with our previous results obtained with pure curcumin [23], but NDS27exhibited greater inhibitory effect than the parent compound curcumin to reduce NADPH oxidase assembly since at 10^−4^ and 5 × 10^−5^ M, it totally inhibited the enzyme activity in cell-free assay while curcumin did not (about 45% of inhibition at similar concentrations [23]). The inhibitory potential of curcumin lysinate salt in cell-free system was somewhere between that of curcumin and NDS27 (data not shown). The excipient for NDS27, HPβCD, showed more efficient inhibitory effect on the activity of NADPH oxidase than NDS27 itself, while NDS28 and its excipient, γ-CD, had no effect suggesting that the type of cyclodextrin is important for the mode of action of the soluble form of curcumin. Besides an evident effect on NADPH oxidase assembly, we have also demonstrated that NDS27 and NDS28 both act upstream in the NADPH oxidase activation pathway by inhibiting the PKCδ activity with IC_50_ values of 1.67 × 10^−5^ M and 1.26 × 10^−5^ M, respectively, these values being much lower than the curcumin IC_50_ value which is about 0.6 mM [21]. Although cyclodextrins had no direct effect on the activity of PKCδ, the results obtained by Western blot analysis showed that HPβCD and NDS27 had an inhibitory effect on the translocation of PKCδ, but not γ-CD and NDS28 suggesting a different behaviour of the soluble molecules due to the cyclodextrin used. The difference between HPβCD and γ-CD is based on the cavity size formed by 7 or 8 dextrose units respectively. Another structural characteristic for HPβCD is that the hydroxyl group was esterified with a propyl group that improved its solubility, as compared to β-cyclodextrins [29]. β-Cyclodextrins are also known to be the most effective sterol-acceptors, due to the diameter of their internal cavity which matches the size of these molecules [30]. With Oil Red O colouration, we determined the ability of NDS27, NDS28 and their respective cyclodextrins to extract the lipid content from the PMNs cell membranes. Due to the sensitivity limitation of this technique, the concentrations of the molecules were increased compared to those usually used. Our results demonstrated that HPβCD and, to a bigger extent, NDS27 were able to reduce the lipid content of the cells while NDS28 and γ-CD had almost no effect. This further underlines the important role played by cyclodextrins.

To better understand the behaviour of the drugs, a set of ESR experiments were conducted, using 5-DSA and 16-DSA probes which have a poor solubility in water and tend to localise in the hydrophobic parts of complex systems [31]. The main idea was first to investigate whether or not incubation of NDS27, or its analog NDS28, influences the membrane fluidity. Furthermore, we also wanted to understand the mechanism of exchange between the drugs and membrane and how it could influence the shape and intensity of ESR signals. According to the ESR data obtained on the HL-60 cellular model, we did not observed any changes in membrane fluidity. However, the binding of both 5- and 16-DSA at the membrane surface, indicated by ESR spectra obtained on pellets from cells pre-incubated with the probes, allows us to successfully demonstrate the capacity of NDS27 and HPβCD to interact with the probes. Our ESR results are clearly in accordance with Oil Red O experiments and indicate that NDS27 and HPβCD readily extracted probes from membranes to the supernatant with a better effect on the 5-DSA than the 16-DSA which is more deeply anchored in the membrane. On the contrary, the use of NDS28 and γ-CD, led to weak ESR signals in supernatants showing their low potential to extract the probes from membranes.

The latter results could be extended to the action of these molecules on the enzymes activity. Undoubtedly, a modification of the lipid environment by NDS27 could be responsible for NADPH oxidase and PKCδ inhibitions. Curcumin may also play a role in this modification as it can bind to the membrane in a surface-associated mode at low concentrations and a trans-membrane mode at higher concentrations [32]. In this case, the drug is anchored to the bilayer by a hydrogen bond near the phosphate group of phospholipids in a manner somewhat similar to cholesterol [32]. We have previously demonstrated that curcumin from NDS27 is easily incorporated into PMNs [24], but NDS28 remained to be tested. By HPLC analyses, we determined that curcumin from NDS28 remained in great part in the extracellular medium (85%) while 65% of curcumin from NDS27 was found within the cells [24]. These results suggest that HPβCD easily exchanges curcumin lysinate for lipid components while γ-CD does not, making HPβCD a better drug delivery agent for curcumin lysinate salt. Moreover, HPβCD has been previously used in complexation with other flavonoids to solubilise and increase their antioxidant capacities by protecting molecules from oxidation by radical species [33]. Yancey et al. (1996) suggested that HPβCD is able to diffuse into the proximity of the plasma membrane, meaning that cholesterol molecules could enter directly into the hydrophobic pocket of the cyclodextrin without the necessity of completely desorbing through the aqueous phase [34]. While the suitability of β-CD and its derivatives to interact with the phospholipids is questioned, the potency of β-CD to preferentially extract cholesterol from cell membranes is broadly recognised [35]. NDS27 appeared to perform more efficiently in the extraction of lipid molecules than HPβCD alone. Owing to the incorporation of curcumin in the apolar cavity of HPβCD, a direct exchange of curcumin for cholesterol could occur. On the contrary, HPβCD will first fill its apolar cavity with water molecules that will be subsequently exchanged for cholesterol [36]. The alteration of lipid distribution by curcumin lysinate [37,38] and HPβCD [39] can affect the activity of membrane proteins and the cell signalling through the alteration of lipid raft systems (cholesterol-rich, detergent insoluble compartment membranes) which participate in the signal transduction mechanism [31]. A recent study by Tsukamoto and co-workers [40] showed that curcumin, at very low concentration, modulates the lipid raft domains by localising between the liquid ordered (l_o_) raft and the surrounding liquid disordered (l_d_) phase causing a domain fusion process. In neutrophils, FcγRIIIB receptors, associated with lipid raft by a glycophosphatidyl inositol (GPI) anchor, are implied in the oxidase activation. Upon stimulation, PKCδ and cytosolic components of NADPH oxidase are recruited to these microdomains and PKC phosphorylates p47phox inducing its conformational change and its interaction with the flavocytochrome b_558_ [41,42]. A final possible mechanism to explain the inhibition of NADPH oxidase activity by the action of curcumin on PKC (Ca^2+^ dependent or not) activation, is its interaction with the Ca^2+^ binding domain and with a stimulatory site located in the kinase domain of the enzyme [43].

In conclusion, our results indicate that both soluble forms of curcumin lysinate have similar effects on the neutrophils’ MPO and ROS scavenging activities. But, depending on the cyclodextrin used, NDS27 and NDS28 have a totally different behaviour regarding NADPH oxidase and PKCδ, two enzymes whose activities are strongly linked to lipid rafts. The choice of cyclodextrin vehicle for curcumin lysinate seems essential to preserve and improve the capacity of the salt to interact with membranes, disturb lipid raft domains and enter into the cytosol, and thus to disrupt the pathways involved in the NADPH oxidase activation and the ROS production. Our study demonstrates that NDS27, together with HPβCD, is a better candidate than NDS28 with γ-CD for the modulation of excessive activation of PMNs. These findings may open up therapeutic perspectives to control equine or human pathologies with excessive inflammatory reactions as already demonstrated in *vivo* in horses [44].
